# Long-Term Surveillance and Management of Urological Complications in Chronic Spinal Cord-Injured Patients

**DOI:** 10.3390/jcm11247307

**Published:** 2022-12-09

**Authors:** Shu-Yu Wu, Jia-Fong Jhang, Hsin-Ho Liu, Jian-Ting Chen, Jian-Ri Li, Bin Chiu, Sung-Lang Chen, Hann-Chorng Kuo

**Affiliations:** 1 Department of Urology, Taipei Tzu Chi Hospital, Buddhist Tzu Chi Medical Foundation, New Taipei City 23142, Taiwan; 2 Department of Urology, School of Medicine, Tzu Chi University, Hualien 97004, Taiwan; 3 Department of Urology, Hualien Tzu Chi Hospital, Buddhist Tzu Chi Medical Foundation, Hualien 97002, Taiwan; 4 Department of Urology, Taichung Tzu Chi Hospital, Buddhist Tzu Chi Medical Foundation, Taichung 42743, Taiwan; 5 Division of Urology, Department of Surgery, Yuanlin Christian Hospital, Changhua 51053, Taiwan; 6 Department of Urology, Taichung Veterans General Hospital, Taichung 40705, Taiwan; 7 Department of Urology, Far Eastern Memorial Hospital, New Taipei City 22000, Taiwan; 8 Department of Urology, Chung Shan Medical University Hospital, Taichung 40201, Taiwan; 9 Department of Urology, School of Medicine, Chung Shan Medical University, Taichung 40201, Taiwan

**Keywords:** spinal cord injury, neurogenic bladder, bladder management, complications

## Abstract

Bladder dysfunction is a common complication after chronic spinal cord injury (SCI). Patients may experience renal function loss, urinary tract infection (UTI), urolithiasis, bladder cancer, and even life-threatening events such as severe sepsis or renal failure. Suitable patient care may prevent UTI and urinary incontinence, decrease medication use, and preserve renal function. As the primary goal is to preserve renal function, management should be focused on facilitating bladder drainage, the avoidance of UTI, and the maintenance of a low intravesical pressure for continence and complete bladder emptying. Currently, several bladder management options are available to SCI patients: (1) reflex voiding; (2) clean intermittent catheterization; (3) indwelling catheterization. The target organ may be the bladder or the bladder outlet. The purposes of intervention include the following: (1) increasing bladder capacity and/or decreasing intravesical pressure; (2) increasing bladder outlet resistance; (3) decreasing bladder outlet resistance; (4) producing detrusor contractility; (5) urinary diversion. Different bladder management methods and interventions may have different results depending on the patient’s lower urinary tract dysfunction. This review aims to report the current management options for long-term bladder dysfunction in chronic SCI patients. Furthermore, we summarize the most suitable care plans for improving the clinical outcome of SCI patients.

## 1. Introduction

Patients with chronic spinal cord injury (SCI) are at increased risks of many urinary tract complications. People with SCI are two to five times more likely to die prematurely than people without SCI. The mortality risk increases with injury level and severity and is the highest in the first year after injury [1]. Common urinary tract complications include renal function loss, urinary tract infection (UTI), urolithiasis, bladder cancer, and catheter-induced injury [1,2,3,4]. All complications are secondary to neurogenic lower urinary tract dysfunction (NLUTD), which results from chronic SCI. The main problems of NLUTD include storage and voiding dysfunctions. Patients often experience low bladder compliance, upper urinary tract damage, urine incontinence, or urine retention, which could lead to further morbidity or mortality. NLUTD also has adverse effects on patients’ daily and social activities, and life quality [5].

Many efforts are made to treat urinary tract dysfunction in SCI patients and avoid the above complications. The primary goal is to preserve renal function. Therefore, management is focused on facilitating bladder drainage, avoiding UTI, and the maintenance of a low intravesical pressure for continence and complete bladder emptying in all kinds of bladder management regimes [6]. To achieve good bladder emptying and continence, patients often depend on catheterization for voiding and require medications or surgery [7,8,9]. However, no procedure is risk-free and suitable for everyone. For example, patients may experience serious complications after bladder augmentation, such as adhesive ileus, urine extravasation, or mortality [9]. Even different bladder management options are related to the risk of UTI [10].

Urological complications are common in patients with chronic SCI. If bladder management is not appropriate, patients with SCI may develop high voiding pressure, low bladder compliance, large post-void residual urine volume, and recurrent urinary tract infections. The long-term surveillance of high-risk SCI patients is thus required to avoid urological complications and improve the quality of life. Since the risk evaluation of different conditions is different, we discuss them below in this review article. Moreover, physical and urological examinations should be performed on SCI patients on a regular basis, depending on their risk of upper urinary tract deterioration. For SCI patients who have received different bladder management regimes and surgical interventions, education on proper bladder emptying and regular surveillance are mandatory. Regular urodynamic studies and upper urinary tract condition evaluations (including hydronephrosis, ureter reflux, and renal function) should be used to determine appropriate bladder management. It is critical to identify high-risk patients in order to prevent renal function deterioration in those with chronic SCI-NLUTD. In order to avoid renal function deterioration and urological complications, the annual active surveillance of bladder and renal function is required, especially in SCI patients at high risk. On the other hand, the patients’ perspective is important; the three most important issues in SCI patients are exercise, nutrition, and pain management, as noted in a previous report that proved that less disturbance caused by bladder management is possible in a well-supported environment [11]. The current evidence and expert opinions on patient-centered bladder management of NLUTD in chronic SCI patients in Taiwan are presented in this article.

## 2. Bothersome Urinary Symptoms, Common Oral Medications, and Bladder Management in Chronic SCI Patients

The level of SCI plays a critical role in patients’ symptoms. Patients with high-level spinal cord (infrapontine–suprasacral) lesions show detrusor overactivity (DO) and detrusor sphincter dyssynergia (DSD), which result in both storage and voiding symptoms. Patients with sacral/infrasacral cord lesions have a hypocontractile or acontractile detrusor, which results predominantly in voiding symptoms [12,13]. In human and animal studies of suprasacral cord lesions, the factors related to DO after SCI can be classified according to the involved organs, including the micturition center, bladder urothelium, and efferent and afferent nerves [14,15]. The umbrella cell layer is damaged after SCI, which leads to reduced transepithelial resistance [16]. Furthermore, long-term changes of the bladder after SCI include increased expression of gap junction proteins, which alter the bladder contractions [17]. The bladder C-fiber becomes mechanosensitive and initiates automatic micturition after SCI [18]. In SCI rats, both Aδ and C-fiber afferents had been reported to promote bladder reflexes [19]. In humans with neurogenic DO, the use of C-fiber neurotoxins can reduce the density of TRPV1 and P2X3 expression, resulting in significant symptom improvement [20,21]. Urinary incontinence is the most commonly reported symptom in patients with SCI and NLUTD [3]. In Hansen et al.’s study, 43% of 236 individuals with SCI experienced urinary incontinence [22]. Blanes et al. reported a higher rate (88%) of urinary incontinence in patients with traumatic paraplegia [23]. According to different urodynamic findings, the possible reasons for urinary incontinence may be urgency incontinence caused by DO or overflow incontinence secondary to detrusor underactivity [7].

Oral medications used in the treatment of lower urinary tract dysfunction in chronic SCI patients include drugs for storage symptoms and voiding symptoms [12]. Antimuscarinic drugs are the first-line option for neurogenic DO; they reduce neurogenic DO-induced incontinence by inhibiting the parasympathetic pathways [24,25,26]. The most commonly reported adverse events are dry mouth and constipation, which often affect patient compliance [27]. Furthermore, the potential risk of developing dementia after antimuscarinic medications has been reported [28]. Beta-3-adrenergic receptor agonist (mirabegron) is another potential treatment option for neurogenic DO without antimuscarinic adverse effects [29,30]. However, there is still no evidence concerning the use of mirabegron as a first-line therapy for neurogenic bladder [30]. The most common adverse effect of mirabegron is the increase in blood pressure, which is positively correlated with the duration of treatment [31]. Therefore, mirabegron is not recommended in those with severe uncontrolled hypertension [32]. Alpha-adrenergic blockers can decrease the bladder outlet resistance effectively [33,34]. The disadvantage and adverse effects of oral medications in SCI patients are summarized in Table 1.

Urine retention is the other common bothersome symptom in chronic SCI patients with detrusor underactivity (DU) or DSD. On the other hand, cholinergic drugs, such as bethanechol and distigmine, have been thought to enhance detrusor contractility. However, currently, there is not enough evidence to support the use of cholinergic medications in underactive urinary bladder [35]. In theory, the use of bethanechol should be avoided in those with DSD and high intravesical pressure to prevent renal function damage. A part of SCI patients with bladder problems often have a lower quality of life and require many other management methods [36,37]. Only 21% of SCI patients reported normal voiding without other bladder management regimes in a cross-sectional survey, and the type of bladder management affected the quality of life. In a previous cross-sectional study of patient-reported outcomes, patients receiving clean intermittent catheterization (CIC) performed by an attendant and those with indwelling catheters were the groups with the worst mental status according to the results of Short-Form 36-Item Health Survey and King’s Health Questionnaire [38]. Currently, there are several bladder managements options available: (1) reflex voiding; (2) CIC; (3) indwelling catheterization (IDC; including urethral or suprapubic catheterization).

### 2.1. Reflex Voiding

Reflex voiding is involuntary emptying, which can be manually induced or spontaneous. Patients with suprasacral cord injury often have detrusor hyperreflexia and DSD, which result in high voiding pressure [39]. Patients with upper motor neuron SCI usually use suprapubic tapping of the bladder to elicit detrusor contraction. The biggest problem of reflex voiding is that patients may not have adequate detrusor contractility to complete bladder emptying [40]. In those who are unable to achieve complete bladder emptying, a combination with CIC may be needed [41]. Some patients without detrusor hyperreflexia to elicit reflex detrusor contractions may use the Credé maneuver, the Valsalva maneuver, or both to facilitate self-voiding in order to empty the bladder [42].

Patients may need fluid restriction and intermittent catheterization. Regular voiding every three hours with post-void residual urine of less than 100 mL has been found to be a good predictor for the discontinuation of catheterization [43]. Reflex voiding has some disadvantages. Persistent and increasing intra-abdominal pressure may exacerbate hemorrhoids, hernia, and vesicoureteral reflux (VUR). On the other hand, adequate bladder emptying made possible by reflex voiding requires a less dyssyneric urethral sphincter, which usually presents in patients with incomplete upper motor neuron lesions. Furthermore, if patients used the Valsalva maneuver to facilitate bladder emptying in addition to reflex voiding, DSD may worsen, leading to difficult urination [44].

### 2.2. Clean Intermittent Catheterization

CIC is the preferred form of management of the bladder when adequate low intravesical pressure and acceptable bladder capacity are achievable. This method avoids the presence of a persistent foreign body and is closer to the physiological condition. Furthermore, it is a reversible and noninvasive treatment compared with other bladder management methods. However, despite the advantages of CIC, it is still not an ideal bladder management option for every SCI patient. A certain part of SCI patients might not maintain CIC over the long term [36]. Many studies have focused on bladder management changes in SCI patients. In Sekar et al.’s [45] study in 1997, 1114 SCI patients used different types of bladder management; the number of patients undergoing CIC dropped from 33% to 5% within 10 years after discharge. In a more recent study by Chen et al. [46] based on data from Taiwan, CIC was more frequently used in patients that had lived with SCI for less than one year, but the CIC rate significantly decreased as the SCI duration increased. Patients that had lived with SCI for more than five years preferred IDC to CIC. This is not specific to one country but is a worldwide phenomenon. Lane et al.’s [47] study of US patients also showed similar results; a total of 47.8% of SCI patients using CIC switched to urethral or suprapubic catheters after 11 years of follow-up. Complications may arise from the practice of CIC, including UTI, urethral stricture, hematuria, and urethral false passage [48]. The most common reasons to discontinue CIC are related to patient preference and physician recommendation. Patients may dislike CIC due to inconvenience during work, if they are tetraplegic, or because they lack a care giver. In the case of frequent UTIs, hydronephrosis, and severe urinary incontinence, the physician may advise the patient to change to IDC.

### 2.3. Indwelling Catheterization

Indwelling catheterization (including urethral or suprapubic catheterization) is widely used in a third of SCI patients. The two most common indications for long-term catheterization are recalcitrant urinary incontinence and urinary obstruction that is not corrected with surgery [49]. The disadvantages of indwelling urinary catheters are almost always related to bacteriuria. From the moment the urethral catheter is introduced, the risk of incidence of bacteriuria is 5% to 10% per day [50,51]. On the other hand, according to the usual daily practice, patients with IDC may experience the infection of cystostomy wound or external meatus even without a systematic report. The possible mechanisms include the following: (1) the catheter acts as a conduit for the entry of microorganisms into the bladder; (2) it provides a medium for bacteria to attach and reduces bacteriuria excretion; (3) as a foreign body, the catheter may affect the integrity of the bladder urothelium, which creates a pathway for microorganism to invade the human body; (4) bacteria may include urease-producing organisms that cause stone formation and encrustation, leading to obstruction, which leads to urine retention and the following cystitis. A recent systematic review compared the effects of catheter-based bladder drainage methods on UTI risk in SCI and neurogenic bladder. It reported that compared with IDC, CIC was associated with a lower rate of UTI. However, the comparisons of urethral catheterization with suprapubic catheterization and of suprapubic catheterization with CIC gave mixed results [10]. In this systematic review, the authors reported that CIC was associated with a lower odds ratio of UTI in five out of six studies. The detailed number of lower rates of UTI was not reported there. In comparing urethral and suprapubic catheterization, SCI patients with a chronic catheter have similar complication rates of UTIs, recurrent bladder/renal calculi, and cancer [52]. Bladder irrigation or washouts have also been reported in the treatment of catheter-related UTI, but the evidence is not adequate to conclude if washouts are beneficial or harmful [53]. Nonetheless, an ideal catheter with sufficient antimicrobial effectiveness is also a possible solution [54]. Urethral and scrotal complications are higher with urethral catheterization. However, the procedures to perform a suprapubic cystostomy could induce some severe morbidities, such as intestinal injury, which offsets the benefits [55].

## 3. Long-Term Complications in Chronic SCI Patients

The common complications of chronic SCI include recurrent UTIs, bladder cancer, renal function loss, urolithiasis, VUR, and catheter-induced injury. The incidence of UTI in SCI patients ranges from 10 to 68% in different studies. This large difference indicates that the incidence of UTI varies widely according to the healthcare setting and patient characteristics [51]. Generally, increased residual urine and decreased bladder compliance may cause patients with SCI to contract UTI [56]. Abnormal lower urinary tract radiological findings, detrusor pressures of ≥75 cm H_2_O, and reduced cystometric bladder capacity (<200 mL) have been found to predict upper urinary tract deterioration in SCI patients [57]. Due to the high catheter-dependence rate, catheter-related UTIs account for the majority of the incidence of UTI in SCI patients. In patients practicing CIC, the mean incidence of UTI is 10.3 cases per 1000 catheter days; after three months, the rate is less than two cases per 1000 catheter days. Once a urethral catheter is in place, the daily incidence of bacteriuria is 3–10%. Most patients have bacteriuria within 30 days [58,59]. Correct and appropriate catheterization is critical to prevent catheter-related UTIs. Frequent checks to evaluate the need to prolong catheterization are indicated. Almost 50% of urinary catheters are placed inappropriately and retained longer than needed [60]. From the moment the urethral catheter is introduced, the incidence of bacteriuria is 5% to 10% per day [43]. Indwelling catheterization has been proved to be one of the risk factors of UTI in chronic SCI patients [51]. In the current longest study reported by Gao et al. [61], recurrent UTI was noted in all patients with a median follow-up of 45 years. The average incidence was 6.1 cases per 5 years per person. Dermatitis is another common complication after chronic infection. The most common dermatological condition in SCI patients is infection, mostly fungal infection [62]. Local fungal infections are commonly found below the level of SCI [63]. Immunological changes, immobile daily activity, and moist perineal conditions could also be the possible reasons, especially in patients with urinary incontinence [64,65].

### 3.1. Bladder Cancer

Patients with SCI are at increased risk of bladder cancer and are more likely to be diagnosed at a later stage. The incidence of bladder cancer in SCI patients is 6‰, and the mean onset time after SCI is 18–34 years in earlier reports [66,67]. A more recent study in 2017 reported by Böthig et al. [68] showed a much lower incidence of bladder cancer in SCI patients (24 out of 6599 patients, 3.6‰). The average age at bladder cancer diagnosis was 57.7 years, which was also below the average for bladder cancer in the local general population. Another report by Michel et al. [69] in 2022 showed that the incidence of bladder cancer in neuro-urological patients was 174.9/100,000 persons/year. The incidence of bladder cancer was 791.1/100,000 persons/year in SCI patients. Indwelling catheters, especially those in place for over 10 years, are thought to be a particular risk for developing bladder cancer [70]. However, more recent studies have reported that more than half of patients developing bladder cancer are without a history of catheterization, suggesting that neurogenic bladder is a more significant cancer risk than indwelling catheter [71]. A special tumor subtype was found in SCI patients with a higher rate of squamous cell bladder cancer. The one-year overall survival rate in this group was 62.1% [71]. A number of diagnostic tools, such as routine cystoscopy surveillance, have been used for early cancer detection in SCI patients. However, there is no general consensus on whether or not routine screening is useful [72,73]. There is even a study reporting that SCI patients who survived their diagnosis of bladder cancer had actually had fewer screening cystoscopies (mean number: 8.6 vs. 18.9) than those who died from the disease [74]. However, we recommend regular urology clinic follow-ups for every SCI patient. The potential benefits of detecting an early malignancy should be balanced against the inconvenience and likely risks associated with screening practices in this population.

### 3.2. Urolithiasis

Urolithiasis is another common condition in patients with SCI. The risk of developing renal stones is estimated to be 7–20% over a 10-year period [75,76]. The risk is also progressive and increases with time; in a long-term follow-up study, 38% of SCI patients developed renal stones [77]. The highest risk of stone formation is in the first 3–6 months after the initial SCI; this is thought to be due to prolonged immobilization resulting in bone resorption and subsequent hypercalciuria [78,79]. The most common forms of urolithiasis in SCI patients are apatite (calcium phosphate) or struvite (magnesium ammonium phosphate) [80]. Commonly reported risk factors include urinary tract stasis, incomplete bladder emptying, chronic catheterization, recurrent UTIs, and immobilization-related hypercalciuria [81]. The current evidence for the relationship between the risk of stone formation and different levels of SCI is contradictory and inconclusive [82]. Patients with upper tract stones are significantly more likely to have had a previous bladder stone than those without. Those with severe injury and requiring instrumentation for bladder emptying are more likely to develop kidney stones [82,83]. Early detection and aggressive treatment of urolithiasis in SCI patients reduce the potential for renal damage.

### 3.3. Vesicoureteral Reflux

VUR is a condition in which urine flows backward from the bladder to the ureter and even the kidney. It usually occurs after prolonged high intravesical pressure [84,85]. Usually, VUR develops within four years from SCI. Recurrent UTIs, hydronephrosis, renal failure, and/or death could result after poor VUR control [86,87]. In addition to direct injury caused by high intravesical pressure, chronic infections also play a role in weakening the valve mechanism by scarring the vesicoureteral junction [88]. Severe trabeculated bladder wall with a diverticulum near the ureteral orifice affects the compression of the submucosal ureteral tunnel [89]. Patients with suprasacral SCI tend to develop VUR due to high filling pressure and bladder outlet obstruction secondary to DO and DSD [90]. Age at onset of SCI, duration of SCI, IDC for more than six months, urodynamic low bladder capacity, high maximum detrusor pressure, recurrent UTI, and absence of antimuscarinic medications have been found to be associated with VUR, as reported previously [87,91,92]. The positive predictive factors for VUR were a high maximum detrusor pressure of ≥75 cm H_2_O, indwelling urethral catheterization, CIC, age ≥ 60 years at onset of SCI, and absence of antimuscarinic medications [87]. These results were compared to those of patients with balanced bladder (the ability to void with acceptable residual urine of less than 100 mL) [87]. Currently, there is no direct evidence on the risk of VUR in different bladder management regimes. However, it can be inferred that increases in intravesical pressure due to reflex voiding is the most likely risk factor for VUR. The prevention and early detection of VUR are important to avoid further irreversible damage in SCI patients.

### 3.4. Loss of Renal Function

The protection of renal function deterioration is always the primary goal of treating neurogenic bladder dysfunction. All the bladder management methods should have this priority. The bladder management methods described above are designed to avoid complications that lead to the impairment of renal function. A long-term follow-up report by Elmelund et al. [93] showed that the cumulative risk of moderate renal deterioration (functional distribution outside 40–60% on renography or relative glomerular filtration rate (GFR) ≤ 75% of the expected rate according to age and gender) was 58% after 45 years of follow-up. The cumulative risk of severe renal deterioration (functional distribution outside 30–70% on renography or a relative GFR of ≤51%) was 29%. Only the dilatation of the upper urinary tract and a renal/ureter stone requiring removal significantly increased the risk of moderate-to-severe renal deterioration. A large study by Fischer et al. [94] showed that older age, female sex, and a non-traumatic cause of injury were associated with increased odds of chronic kidney disease (CKD), and black people and a duration of injury of ≥10 years were associated with decreased odds of CKD. Renal examinations should be carried out regularly, especially in those using non-recommended bladder management methods and/or patients presenting with upper urinary tract dilatation.

## 4. Complications after Common Interventions for Lower Urinary Tract Symptoms in Chronic SCI Patients

As mentioned above, patients with SCI often depend on catheterization for voiding. Therefore, catheter-related injuries may occur in some individuals. The reasons for and risk factors of catheter-related urethral injury include the lack of sensation in the urethra, trauma to the urethra during previous catheterizations, pelvic floor muscle and urethral sphincter spasms, and previous bladder outlet operations [95]. Vírseda-Chamorro et al. [96] analyzed SCI and urethral diverticulum and reported that the age of onset of the spinal injury, the sphincterotomy procedure, a personal history of UTI, and chronic need for either IDC or external condom drainage were associated risk factors. A further analysis showed that IDC was the only risk factor of urethral injury.

Many interventions have been attempted to solve the voiding problems of chronic SCI patients. Unfortunately, every intervention has its potential complications. Nevertheless, the operative interventions for bladder management and urological complications can be executed according to their purposes and target the bladder or the bladder outlet. The purposes of these procedures include the following: (1) increasing bladder capacity and/or decreasing intravesical pressure; (2) increasing bladder outlet resistance; (3) decreasing bladder outlet resistance; (4) producing detrusor contractility; and (5) urinary diversion. We summarize the current surgical procedures to achieve the goals of bladder management methods in chronic SCI patients in Table 2 according to previous reports [39,97].

### 4.1. Increasing Bladder Capacity and/or Decreasing Intravesical Pressure

In SCI patients with severe DO, high intravesical pressure is often induced, resulting in low bladder compliance and severe incontinence. Recent studies have shown that intradetrusor injection of onabotulinumtoxinA (botulinum neurotoxin serotype A (BoNT-A)) has high therapeutic efficacy in treating DO in SCI patients [98,99]. Patients’ quality of life can be significantly improved with repeated injections of BoNT-A to reduce DO and improve bladder compliance [100]. However, the procedure does not have a permanent effect; patients need repeat treatment to maintain the therapeutic efficacy [101]. Urinary retention is inevitable after detrusor BoNT-A injections, and temporary CIC for bladder emptying might be needed, which decreases patients’ intention of repeating treatment [102,103]. Except direct injection, urothelial denudation with protamine sulfate or dimethyl sulfoxide, liposome encapsulated BoNT-A, and other physical approaches are being studied [104]. Liposome encapsulated BoNT-A was reported to be safe and effective in enhancing toxin activity while reducing its toxin degradation [105]. Even if this treatment successfully reduced urinary frequency and urgency, it did not significantly reduce urgency urinary incontinence episodes [105].

In addition to intravesical BoNT-A injections, other more invasive and irreversible procedures, including bladder autoaugmentation and augmentation enterocystoplasty, are available. Bladder autoaugmentation involves complete myomectomy of the bladder dome to form a wide-open artificial bladder diverticulum. In enterocystoplasty, a short bowel segment is used to form a pouch. Common disadvantages and complications include the need for CIC, recurrent UTIs, urinary tract stones, and chronic diarrhea [9]. Because the operation is irreversible and involves other organs, both patients and doctors should carefully consider the available options before embarking on the procedure.

### 4.2. Increasing Bladder Outlet Resistance

Sling surgery and periurethral collagen injection are options for SCI patients with severe urine incontinence due to intrinsic sphincter deficiency. Sling surgery can restore urethral support and compress the urethra to avoid urine leakage. The sling may be constructed with fascial tissue or a synthetic mesh. Fascial slings are more cost-effective and have lower rates of bladder perforation or urethral erosion than slings made with synthetic materials. Periurethral collagen injections produce a direct bulking effect on the urethral lumen and cause obstruction [106]. Sling surgery has some disadvantages. As a foreign body, a sling may not be able to remain still and function in the location where it was placed. The main symptoms for sling revision are severe dysuria, followed by urinary retention or severe wound discomfort [107]. Furthermore, bladder or urethral injury may occur during surgery; mesh exposure has also been mentioned in some studies [108,109]. Pain, UTIs, pseudocyst formation, and urine retention are the most common complications after the injection of urethral bulking agents. In previous studies, the overall complication rate was 32%, and most cases required noninvasive treatment [110,111].

In male patients with neurogenic bladder and urinary incontinence, artificial urinary sphincter surgery is considered the gold standard of treatment. The most consistently studied artificial urinary sphincter, AMS 800, confers a high rate of post-surgery continence (70–92%). The most common disadvantages are infection, erosion, and a high rate of repeat surgery [112]. In Hebert et al.’s [113] study, more than 20% of patients underwent revision surgery for mechanical failure or urethral atrophy with a median follow-up of 4.1 years. Revision surgery was associated with significantly increased cumulative incidence of infection, urethral erosion, mechanical failure, and urethral atrophy.

### 4.3. Decreasing Bladder Outlet Resistance

BoNT-A can also be used in patients with DSD to decrease sphincter muscle tone. It can facilitate self-voiding and make CIC easier [114]. BoNT-A can be injected into the urethral external sphincter via both the transurethral and transperineal approaches. Currently, no standardized technique exists, but 100 units BoNT-A is suggested in most reports [115]. External sphincterotomy has been used in clinical practice for many years. Sphincterotomy can improve dysreflexia symptoms and residual urine volume and reduce the rate of UTI [116,117]. However, a high failure rate (>50%) has been reported, including urethral stricture, incomplete sphincterotomy, and postoperative bladder hypocontractility [117,118]. A significant number of men with SCI after the procedure continue to have high intravesical pressure, raised leak point pressure, recurrent UTI, or severe autonomic dysreflexia [119]. The endoscopic insertion of a urethral stent can provide an open urethra in patients with DSD. It helps to reduce the residual urine volume and autonomic dysreflexia. Although it is a reversible procedure, a high failure rate and an increased infection rate are serious flaws that make it a less attractive option [120]. The transurethral incision of the bladder neck is regarded as a more controversial method. The incision of the bladder neck provides lower bladder outlet resistance and preserves the external sphincter, thus enabling the avoidance of total incontinence [121,122]. Most of the information about the transurethral incision of the bladder neck comes from male patients receiving transurethral resection of the prostate. As evidence of its efficacy in SCI patients is still lacking, it should be used carefully.

### 4.4. Producing Detrusor Contraction

Currently, sacral anterior root stimulation is the only way with evidence to produce detrusor contraction in highly selected patients [123,124]. Since its stimulation amplitude exceeds the pain threshold, this technique can only be used for complete lesions above the implant level. Although the urethral sphincter is also stimulated by nerve root stimulation, the striated muscle relaxes faster than the detrusor smooth muscle. Patients experience post-stimulus voiding [125]. It has been shown that the detrusor pressure decreases over time, but the changes do not seem to be clinically relevant during first decade after the surgery [126]. Defects of the stimulator cables or the receiver plate are the most common complications requiring surgical revision. Stimulation failure, liquor leakage, and infection have also been reported [123].

### 4.5. Urinary Diversion

Urinary diversion refers to a detubularized reconstructed bowel with a continent catheterizable abdominal stoma or conduit for continuous urine drainage. Urinary diversion is indicated for SCI patients who have a destroyed bladder outlet or are unable or unwilling to manage IDC or CIC. Incontinent techniques are preferred for urinary reconstruction. The incontinent diversion should be performed in patients with poor renal function and is not suitable for catheterization [127]. The discussion of complications is mostly centered on augmentation enterocystoplasty. Due to their similar surgical principles, both procedures share the same complications. Commonly reported minor complications include hydronephrosis, recurrent UTIs, and chronic diarrhea. In addition, major complications have also been reported, including mortality, drainage failure, urolithiasis, adhesion ileus, and difficult catheterization [9,128,129]. According to the systematic review reported by Hoen et al., bladder augmentation seems to be a highly effective procedure in highly selective patients to protect renal function and to improve the quality of life. However, due to the lack of high-quality evidence, it is difficult to make strong recommendations [130]. Patients undergoing urinary diversion tend to be older and have higher-level SCI, severe autonomic dysreflexia, and greater dependency on healthcare services. In other words, these are the last-line treatments that should be used only in patients with more serious lower urinary tract conditions.

## 5. Summary and Recommendations

Patients with chronic SCI experience different voiding dysfunctions, and the urinary tract condition often progresses over time. Many efforts have been made to protect patients’ urinary function and avoid complications. Catheterization and surgical interventions are commonly used methods. The reported long-term complications in SCI patients include recurrent UTIs, bladder cancer, renal function loss, urolithiasis, VUR, and catheter-induced injury. Although patients with NLUTD can be properly diagnosed and treated, all patients should be monitored for the rest of their lives to avoid the development of urological complications and unwanted lower urinary tract symptoms. Patients with the following conditions should be considered to be high risk and require special attention during follow-up surveillance (Table 3) [131,132,133]. Different bladder management methods and interventions also lead to different complications. There is no optimum management regime, only the most suitable method for patients with chronic SCI. The bladder management method depends on the patients’ situation, and a rolling plan of bladder management is needed for SCI patients. To avoid complications and improve life quality, regular follow-ups and urological examinations are necessary.

## Figures and Tables

**Table 1:** Summary of oral medications for neurogenic lower urinary tract dysfunction in chronic SCI patients.

Oral Medication	Disadvantages	Adverse Events
For storage symptoms		
Antimuscarinics	Decrease in detrusor contractility and urine retention	Dry mouth, blurred vision, and constipation
Beta-3 adrenoceptor agonist	Constipation	Tachycardia and hypertension
For voiding symptoms		
Bethanechol	Increase in urine incontinence and profuse salivation	
Alpha-adrenergic blockers	Increase in urine incontinence	Hypotension and nasal stuffiness

**Table 2:** Treatments and surgical procedures for neurogenic lower urinary tract dysfunction in chronic spinal cord-injured patients.

Aim of Treatment	Interventional Procedure	Specific Indications
**To improve continence**	Detrusor BoNT-A injection	DO with/without DSD, AD
	Bladder augmentation	Low compliant bladder, AD
	Autoaugmentation	Low compliant bladder
	Kock pouch diversion	Contracted bladder, severe ISD, high-grade VUR, urethral fistula or stricture
	Suburethral sling	DA/DU with ISD
	AUS implantation	DA/DU with ISD
	Antimuscarinic drugs	DO, with/without DSD
**To facilitate voiding**	Urethral Botox injection	DSD, AD
	TUI-BN	DA/DU, AD, BND
	TUI-P/TUR-P	DA/DU, DO with BND, AD
	External sphincterotomy	Quadriplegia, DSD, AD
	Alpha-blocker, Baclofen	DSD, AD
**For urinary incontinence and bladder emptying**	Continent cystostomy	Paraplegia, DU, and DSD
	CISC/CIC	Paraplegia, good hand function, DU, or DO +DSD
	Cystostomy	DA/DU or DO + DSD
	Indwelling urethral catheter	Quadriplegia, DA/DU, or DO + DSD
**For autonomic dysreflexia**	Ileal conduit diversion	Quadriplegia, DO, DSD, severe AD, severe ISD
	Cystostomy, indwelling urethral catheter	Quadriplegia, DO, DSD, severe AD, severe ISD
	Medications	AD and DSD

BoNT-A, botulinum neurotoxin serotype A; DO, detrusor overactivity; DSD, detrusor sphincter dyssynergia; AD, autonomic dysreflexia; ISD, intrinsic sphincter deficiency; VUR, vesicoureteral reflux; DA, detrusor acontractile; DU, detrusor underactivity; AUS, artificial urinary sphincter; TUI-BN, transurethral incision of the bladder neck; BND, bladder neck dysfunction; TUI-P, transurethral incision of the prostate; TUR-P, transurethral resection of the prostate; CISC, clean intermittent self-catheterization; CIC, clean intermittent catheterization.

**Table 3:** Classification of risk and follow-up surveillance in patients with chronic spinal cord injury to avoid urological complications.

Risk Stratification	Follow-Up Surveillance
**Low risk:** Normal or stable renal function. Normal upper urinary tract (no hydronephrosis, no stone, no VUR, etc.). PVR ≤ 33% of bladder capacity. Spontaneous voiding with low intravesical pressure and small PVR. No bladder outlet obstruction.	No absolute active surveillance. Annual surveillance if new occurrence of LUTS, UTI, stone, VUR, upper urinary tract change, or renal function deterioration.
**Moderate risk:** Normal or stable renal function. Normal upper urinary tract. Large PVR, incomplete voiding, or urinary retention. DO with high intravesical pressure. Presence of bladder outlet obstruction (including DSD). Recurrent UTIs.	Annual surveillance: history, physical examination, and renal function. Urinalysis and culture for UTI. Upper urinary tract imaging. Urodynamic study or VUDS for new LUTS or renal function deterioration.
**High risk:** Abnormal or unstable renal function. Abnormal upper urinary tract (hydronephrosis, renal stone, VUR, renal parenchymal loss, etc.). DU with large PVR. High intravesical pressure and low bladder compliance. Severe bladder outlet obstruction (including DSD). Recurrent febrile UTIs.	Annual surveillance: history, physical examination, and renal function. Urinalysis and culture for UTI. Upper urinary tract imaging. Urodynamic study or regular VUDS till bladder and renal dysfunction are stable.

VUR, vesicoureteral reflux; PVR, post-void residual volume; LUTS, lower urinary tract symptoms; UTI, urinary tract infection; DO, detrusor overactivity; DSD, detrusor sphincter dyssynergia; VUDS, video-urodynamic study; DU, detrusor underactivity.

## Data Availability

Not applicable.
